# Imaging tumor microscopic viscosity in vivo using molecular rotors

**DOI:** 10.1038/srep41097

**Published:** 2017-01-30

**Authors:** Lyubov’ E. Shimolina, Maria Angeles Izquierdo, Ismael López-Duarte, James A. Bull, Marina V. Shirmanova, Larisa G. Klapshina, Elena V. Zagaynova, Marina K. Kuimova

**Affiliations:** 1 Institute of Biomedical Technologies, Nizhny Novgorod State Medical Academy, Minin and Pozharsky Square, 10/1, Nizhny Novgorod, 603005, Russia; 2 Institute of Biology and Biomedicine, Nizhny Novgorod State University, Gagarin Avenue, 23, Nizhny Novgorod, 603950, Russia; 3 Department of Chemistry, Imperial College London, South Kensington, London SW7 2AZ, UK; 4 Razuvaev Institute of Organometallic Chemistry RAS, Tropinina Street, 49, Nizhny Novgorod, 603950, Russia

## Abstract

The microscopic viscosity plays an essential role in cellular biophysics by controlling the rates of diffusion and bimolecular reactions within the cell interior. While several approaches have emerged that have allowed the measurement of viscosity and diffusion on a single cell level *in vitro*, the *in vivo* viscosity monitoring has not yet been realized. Here we report the use of fluorescent molecular rotors in combination with Fluorescence Lifetime Imaging Microscopy (FLIM) to image microscopic viscosity *in vivo*, both on a single cell level and in connecting tissues of subcutaneous tumors in mice. We find that viscosities recorded from single tumor cells *in vivo* correlate well with the *in vitro* values from the same cancer cell line. Importantly, our new method allows both imaging and dynamic monitoring of viscosity changes in real time in live animals and thus it is particularly suitable for diagnostics and monitoring of the progress of treatments that might be accompanied by changes in microscopic viscosity.

The microscopic viscosity is one of the key parameters that controls the diffusion rate of molecular species and hence affects the reaction rates of diffusion controlled processes on the microscopic level. Therefore, the microscopic viscosity plays an extremely important role in the functioning of a healthy cell, and abnormal levels of viscosity on both the cell and the organism level have been linked to disease and malfunction^1^,^2^,^3^,^4^,^5^,^6^,^7^. It follows that it is extremely attractive to be able to map the distribution of microscopic viscosity on a single cell level^8^. Consequently a plethora of fluorescence-based methods have been developed that allowed the determination of diffusion coefficients or viscosity in biological systems with diffraction-limited resolution, such as fluorescence correlation spectroscopy (FCS)^9^,^10^,^11^, fluorescence recovery after photobleaching (FRAP)^12^, fluorescence steady state and time resolved anisotropy^13^,^14^,^15^, and fluorescence imaging of molecular rotors^8^,^16^, to name a few. These methods were successfully used for the determination of diffusion coefficients and viscosity in live cell and in a large variety of model lipid systems. Notably, fluorescent molecular rotors allowed both the spatially resolved quantitative imaging of viscosity, down to the resolution of individual cell organelles, and the dynamic measurements of viscosity in real time^8^. These two attractive features set molecular rotor-based imaging apart from other available methods used for viscosity determination, which typically only allow single point measurements to be performed.

Molecular rotors are small synthetic viscosity-sensitive fluorophores in which fluorescence parameters are strongly correlated to the microviscosity of their immediate environment^8^,^16^. Importantly, fluorescence ratiometric and lifetime detection from molecular rotors allowed to overcome difficulties associated with an unknown fluorophore concentration and thus enabled quantitative viscosity mapping to be performed. These measurements provided the wealth of biologically relevant information on model lipid membranes^17^,^18^,^19^, bacterial^20^,^21^,^22^ and eukaryotic cells and cellular organelles^23^,^24^,^25^,^26^,^27^,^28^,^29^,^30^,^31^,^32^,^33^, and allowed viscosity monitoring during lipid (photo)oxidation^34^, cell death^31^, bacterial sporulation and deactivation^20^,^21^, and bacterial membrane viscosity changes in response to variations in temperature^22^. While the fluorescence-based approaches, including molecular rotors, allowed the measurements of viscosity and diffusion on a single cell level *in vitro*, the *in vivo* viscosity monitoring has not yet been realized.

The aim of the present work was to develop a method that allows obtaining microscopic viscosity maps from individual cancer cells and connecting tissue *in vivo*, in a mouse tumor model. The viscosity mapping was performed based on the fluorescence lifetime monitoring of molecular rotors that were previously shown to be effective reporters of the microscopic viscosity *in vitro*. We investigated the kinetics of accumulation and clearance of the dyes from subcutaneous tumors *in vivo* and developed a delivery formulation that allowed quantitative viscosity imaging and monitoring viscosity changes over prolonged periods of time in an animal model.

## Results

### Spectroscopic characterization of molecular rotors

**Figure 1:**
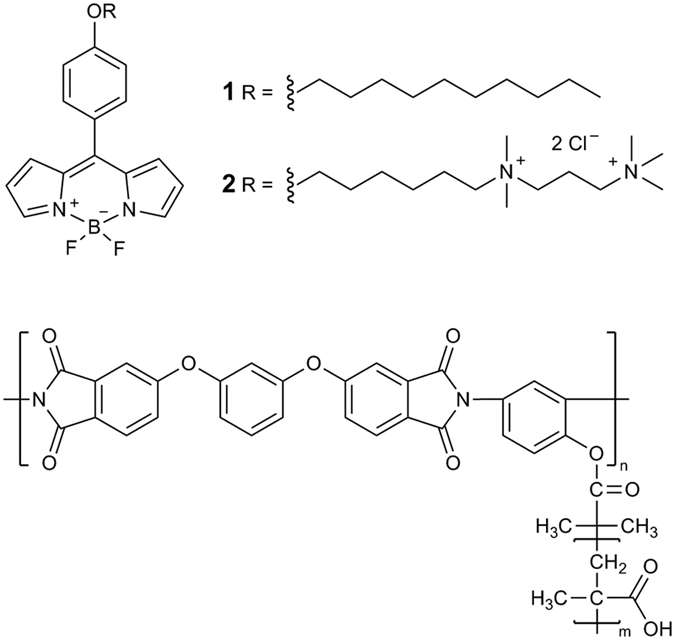
The molecular structures of molecular rotors BODIPY1, BODIPY2 and a polymeric brush used for solubilisation of BODIPY1.

BODIPY (4,4-difluoro-4-bora-3a,4a-diaza-*s*-indacene) molecular rotors have already demonstrated their usefulness as versatile probes suitable for viscosity determination in a variety of *in vitro* micro-heterogeneous systems. Of particular relevance to this work is their use as viscosity probes for cellular organelles, including the plasma membrane of live cells^8^,^29^. To this end, molecular rotor BODIPY2^29^ (Fig. 1) allowed selective staining of the plasma membrane of eukaryotic cells by avoiding efficient endocytosis, typical of BODIPY1^32^,^33^. All BODIPY rotors reported to date are characterized by good cellular uptakes as well as negligible dark toxicity and photo-toxicity to cultured cells^8^,^29^. BODIPY rotors also possess excellent dynamic range of fluorescence lifetimes for FLIM-based viscosity determination^8^,^29^ that was also demonstrated to be temperature-independent^35^. For this study we chose two molecular rotors, BODIPY1 and BODIPY2, (Fig. 1) that were previously characterized as reliable viscosity probes in model membranes^17^,^19^ as well as in plasma membranes of live cultured cells^29^,^32^,^33^.

We note that BODIPY molecular rotors are mostly hydrophobic molecules and display extremely poor water solubility (with the exception of BODIPY2). An intravenous injection of BODIPY1 in water (5% DMSO) to tumor-bearing mice does not result in fluorescence in the animal body (Figure S1). Therefore a solubilising agent is required for delivery of BODIPY1 *in vivo*. We have first investigated the photophysics of BODIPY1 in the presence of increasing concentrations of solubilizing polymeric brushes (the structure shown in Fig. 1) that were previously reported to aid *in vivo* tumor delivery of porphyrin based drugs^36^.

As stated above, BODIPY1 displays a very poor aqueous solubility and is heavily aggregated in aqueous medium, leading to broadened absorption bands, extremely low quantum yield of green fluorescence centred at 515 nm and an appearance of a red emission band centred at 650 nm typical of aggregated species (Figure S2). We have recorded absorption and fluorescence spectra of BODIPY1 in the presence of increasing concentration of polymeric brushes, and as a function of elapsed time after the mixing of the BODIPY1 and the brushes solutions. We found that 1:1 [BODIPY1]:[brushes unit] ratio allows full disaggregation of BODIPY1 after 24 h of mixing, characterized by a sharp absorption band and an intense fluorescence band, typical of a monomeric BODIPY, and a complete disappearance of the red emission band typical of aggregates, Figure S2.

For *in vitro* experiments, we have tested three different incubation conditions on cultured cells: (i) BODIPY1 with low concentration of brushes that allows solubilisation of BODIPY1 but with some spectroscopic features of aggregation present (equivalent to 10:1 [BODIPY1]:[brushes unit]); (ii) BODIPY1 with high concentration of brushes enabling complete BODIPY solubilisation in an aqueous solution and full disaggregation (equivalent to 1:1 [BODIPY1]:[brushes unit]); and (iii) BODIPY2 in water.

### *In vitro* imaging of microscopic viscosity of CT26 cells

**Figure 2:**
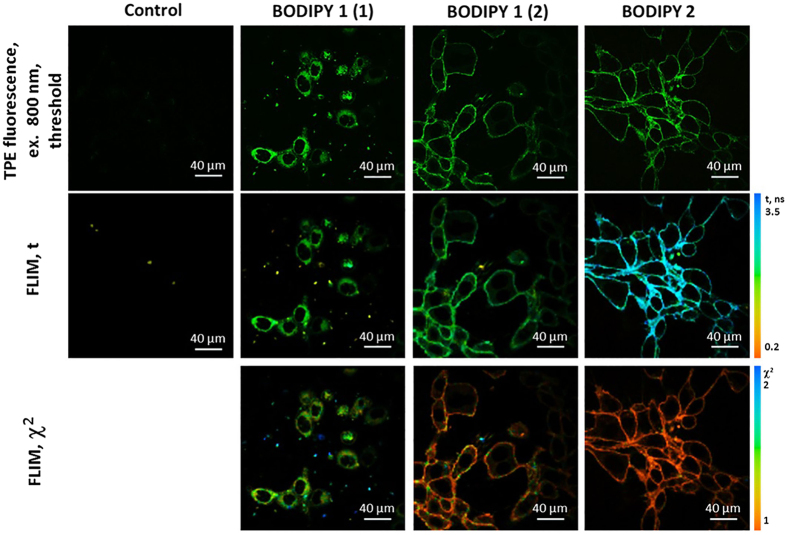
Two-photon excited (TPE) fluorescence and FLIM images of CT26 cells incubated with 4.5 μM solutions of BODIPY1 and BODIPY2. BODIPY1 was dissolved in high and low concentration of polymeric brushes (2 and 12.4 mg/ml, corresponding to labels (1) and (2), respectively). BODIPY2 was dissolved in PBS. Excitation was 800 nm, detection −409–680 nm. Control CT26 cells imaged at identical conditions that were not incubated with any fluorescence dyes are also shown in the first column. The χ^2^ maps confirm good monoexponential fitting in the areas where χ^2^  ≈ 1 (indicated by the orange colour).

We have first incubated murine colon carcinoma CT26 cultured cells with solutions of BODIPY1 and BODIPY2 as described above, Fig. 2. In all cases bright fluorescence typical of BODIPY was observed from cultured cells. FLIM images were recorded using 800 nm pulsed excitation and 409–680 nm detection. While condition (i) produced internally stained cells with punctate fluorescence distribution, incubation conditions (ii) and (iii) produced a clear plasma membrane staining. While the plasma membrane staining upon incubation with BODIPY2 at low temperature was expected, as reported previously^29^, our data shows that the addition of a high concentration of polymeric brushes prevents endocytosis of BODIPY1 into cells, even following an incubation at room temperature.

It is well known that the time resolved fluorescence decays of a monomeric form of BODIPY rotors, when placed in a homogeneous environment, is characterized by monoexponential decays^19^,^29^,^33^. The presence of aggregates, on the other hand, causes the biexponential decay kinetics and renders lifetime/viscosity calibration unusable due to quenching of the viscosity-sensitive monomer BODIPY by aggregates^19^. While the decays recorded from CT26 cells following incubation with solutions (ii) and (iii) were monoexponential, condition (i) produced biexponential fluorescence decays. This could be seen from high χ^2^ value of a monoexponential fitting, Fig. 2, while the χ^2^ value for a biexponential fitting was close to 1. It is therefore likely that a high concentration of the rotor was delivered to cells in a polymeric brush in the condition (i), causing BODIPY1 aggregation inside cells.

We converted FLIM maps to viscosity maps for conditions (ii) and (iii) only, using previously reported calibrations^17^. According to the calibration curves, the membrane viscosity in CT26 cells was 184 ± 11 and 377 ± 27 cP, respectively. Although it is known that the rotors BODIPY1 and BODIPY2 occupy a similar position in the lipid bilayer^17^, the viscosity recorded by BODIPY1 in the presence of brushes is significantly lower. At the same time it is clear that BODIPY1 dissociated from the brush as its lifetime was significantly shorter than that recorded in a polymeric brush incubation solution (Figure S3). Therefore, from our data it appears that the polymeric brush alters the structure of the plasma membrane, making it less viscous. Changes in lipid ordering upon interaction with polymers were in fact previously reported for other polymers^37^,^38^. Thus, care must be taken when interpreting the results of *in vitro* and *in vivo* imaging using solubilising delivery vehicles that might alter the system under study^39^.

### The accumulation and clearance of molecular rotors *in vivo*

**Figure 3:**
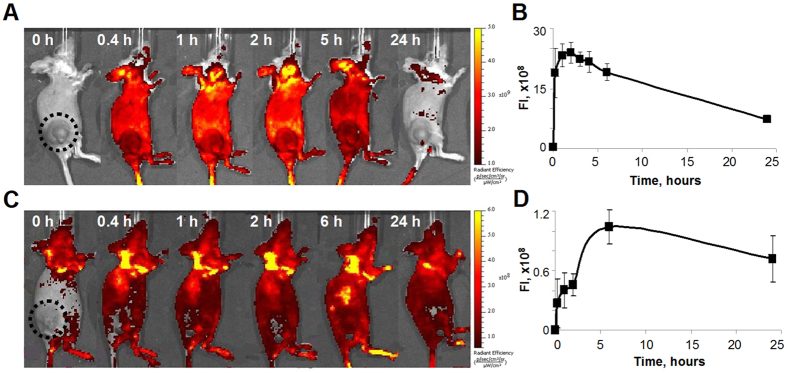
Monitoring the accumulation of molecular rotors BODIPY1 and BODIPY2 in CT26 tumor *in vivo*. Fluorescence images of mice (**A**,**C**) and kinetics of fluorescence in tumors (**B**,**D**) after injection of BODIPY2 at 10 mg/kg (**A**,**B**) or BODIPY1 at 5 mg/kg dissolved in polymeric brushes (at 12 mg/kg) (**C**,**D**). Excitation was 500/30 nm, emission was 540/20 nm. Mean ± SD, n = 4 mice per group.

Having assured that both BODIPY1 and BODIPY2 interact with cultured CT26 cells at incubation conditions (i)-(iii) we next investigated the accumulation and clearance of these two dyes from subcutaneous tumors in mice. We note that intravenous injections of BODIPY1 without polymeric brushes (8 mg/kg; 5% DMSO) does not result in any fluorescence in the tumor bearing mice, Figure S1. Administration of BODIPY2 in water and BODIPY1 dissolved in polymeric brushes at the condition (i) led to increase of the fluorescence intensity in the tumor, indicating accumulation of the rotors in the tumor tissue, however, without pronounced selectivity (Fig. 3). BODIPY2 displayed the maximum fluorescence signal in the tumor in the period from 15 min to 6 hours after an intravenous injection. BODIPY1 reached its maximum tumor uptake as judged by fluorescence by 6 hours post-injection. At 24 hours after administration both rotors remained in the tumor at a relatively high concentration.

Detailed biodistribution study, based on fluorescence of tissue samples *ex vivo*, revealed that the highest content of the rotors was in organs responsible for excretion of the drug from the body – colon, liver and skin. In the case of BODIPY2, kidneys were also involved in the clearance of the drug at a 24 hours’ time-point. In other tissues the concentration of BODIPY2 decreased in the following order: tumor > lungs > muscles > heart > spleen (Figure S6). For BODIPY1 the rank order was muscles > tumor > heart > kidneys > lung > spleen (Figure S5).

**Figure 4:**
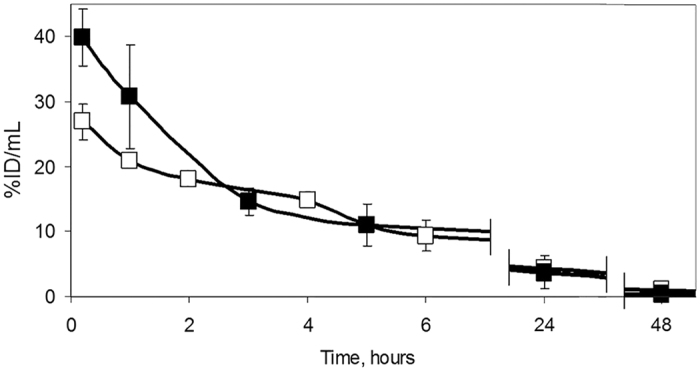
Time course of concentrations of BODIPY2 (■), at 10 mg/kg, and BODIPY1 at 5 mg/kg, dissolved in polymeric brushes (at 12 mg/kg) (□), expressed as percentage of injected dose per mL of plasma after intravenous injection into Balb/c mice with the subcutaneous CT26 tumor. Mean ± SD, n = 4 mice per group.

Analysis of kinetics of BODIPY2 and BODIPY1 in the blood plasma showed gradual decrease of the concentration with complete elimination from the bloodstream by 48 hours post-injection (Fig. 4). It should be mentioned that no acute toxicity effects were observed in mice for any of the injected substances.

### Viscosity imaging in CT26 subcutaneous tumors *in vivo*

**Figure 5:**
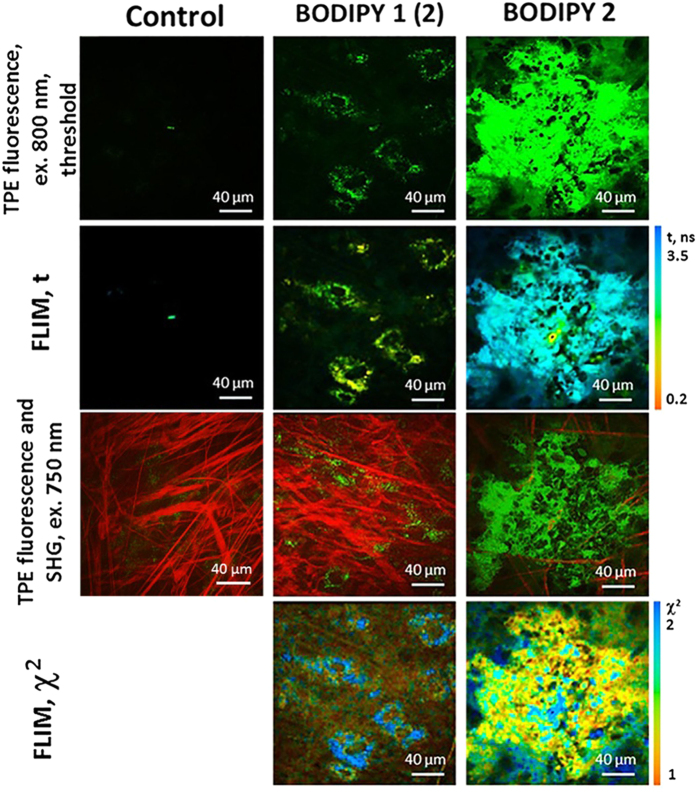
Representative *in vivo* two-photon excited (TPE) fluorescence and FLIM images of CT26 tumor at 40 min after an i.v. injection of BODIPY1 dissolved in polymeric brushes (3 mg/kg BODIPY1 in 8 mg/kg brushes) and at 60 min after an i.v. injection of BODIPY2 (3 mg/kg). Excitation was 800 nm, detection 409–680 nm. The contribution from autofluorescence was low following 800 nm excitation. The second-harmonic generation (SHG) signal from connective tissue (collagen fibers, red) and TPE fluorescence (green) are also shown, following 750 nm excitation. Control CT26 tumor without BODIPY injection imaged at identical conditions is also shown in the first column. The 750 nm excitation leads to autofluorescence in control cells. The χ^2^ maps confirm good monoexponential fitting in the areas where χ^2^ ≤ 1.5 (indicated by the orange to green colour).

Next, we recorded FLIM images of subcutaneous tumors in mice, following injection of BODIPY1 and BODIPY2. All injection conditions produced bright two photon excited fluorescence and FLIM images, Fig. 5. However, as was the case with cultured cells, the condition (i) produced FLIM images that were characterized by biexponential decays of the rotor in both tumor cells and in connective tissue. Furthermore, the short lifetime component of the decay became shorter as a function of elapsed time after the probe injection (Figure S4), giving evidence that the biexponential decay is indeed caused by the dye aggregation that became more prominent with increased time due to the dye accumulation in the tumor. The recorded lifetimes were therefore not converted to viscosity since the calibration curve is not applicable in the presence of aggregated species.

Interestingly, condition (ii) produced biexponential decays in tumor cells but monoexponential decays in tumor connective tissue (collagen), characterized by the fluorescence lifetime of 2.24 ± 0.06 ns, corresponding to a viscosity value of 265 ± 16 cP, Fig. 5. Again, these decays were significantly shorter than those recorded in a polymeric brush incubation solution itself (Figure S3), providing firm evidence that the rotor dislodged from the brush that worked as a delivery vehicle to tumor.

**Figure 6:**
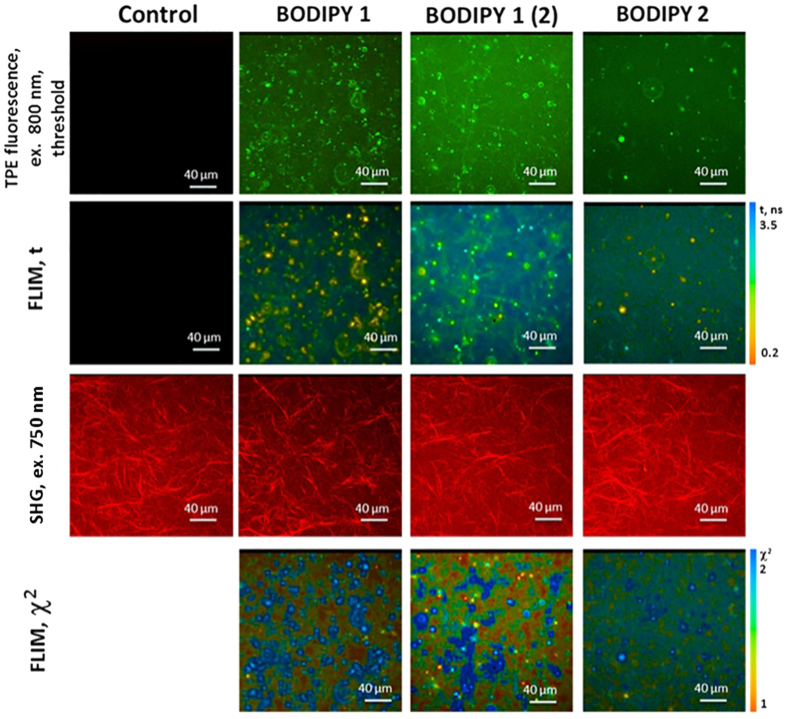
Two photon excited (TPE) fluorescence and FLIM images of cellularized collagen fibres incubated with 45 μM solutions of BODIPY1 (0.5% DMSO), BODIPY1 with polymer brushes (12.4 mg/ml, label (2)) and BODIPY2. Images were recorded using 800 nm excitation and 409–680 nm detection. Control collagen without the addition of BODIPY imaged at identical conditions is also shown in the first column. SHG signal from collagen fibers is shown in red and images were obtained using 750 nm excitation. The χ^2^ maps confirm good monoexponential fitting in the areas where χ^2^ ≈ 1 (indicated by the orange colour). The aggregates of the rotors in collagen samples display higher χ^2^ values.

To ensure that polymeric brushes administered *in vivo* do not affect the viscosity of collagen, we measured fluorescence lifetime of BODIPY1 and BODIPY2 in collagen hydrogels *in vitro*, Fig. 6. We have tested BODIPY1 at the condition (ii) and BODIPY2 at the condition (iii) and also measured pure BODIPY1 dissolved in 0.5% DMSO as a polymeric brush-free control. BODIPY2 at the condition (iii) showed biexponential decays in collagen, similarly to what was observed *in vivo*, probably due to its aggregation in this media. However, monoexponential decays were observed for BODIPY1 at both conditions tested (Fig. 6). BODIPY1 in the presence of polymeric brushes displayed the fluorescence lifetime of 2.79 ± 0.15 ns, corresponding to a viscosity value of 429 ± 51 cP. For BODIPY1 administered without polymeric brushes fluorescence lifetime was 2.22 ± 0.06 ns, corresponding to viscosity 260 ± 15 cP. Therefore, the viscosity of collagen phantoms can be measured by BODIPY1 *in vitro* but not by BODIPY2. Furthermore, as in the case of cultured cells, the measured viscosity of collagen *in vitro* was affected by the delivery of the rotor in the polymeric brushes, giving higher viscosity in the presence of brushes. However, the value obtained for collagen in the tumor *in vivo*, closely corresponded to that of the polymeric brush-free collagen phantoms. Thus we conclude that polymeric brushes do not alter the viscosity of connective tissue *in vivo*, yet they enable BODIPY1 delivery.

An injection of water-soluble BODIPY2 into the tail vein of tumor bearing mice (condition iii) produced monoexponential fluorescence decays in the tumor cells, characterized by the fluorescence lifetimes of 2.67 ± 0.06 ns, Fig. 5. The values recorded in tumor cells *in vivo* match the fluorescence lifetimes recorded in CT26 cultured cells (Fig. 2) and thus indicate the same viscosity as was recorded in cells *in vitro*, 386 ± 19 cP. *In vivo* monitoring of the viscosity from 20 to 80 min after BODIPY2 administration showed no changes of the viscosity within this period of time (Figure S4). Subsequent measurements of BODIPY2 fluorescence lifetime in 24 hours in the same tumors displayed biexponential decay, probably associated with redistribution of the rotor in tumor cells and its aggregation. The results of viscosity measurements *in vivo* and *in vitro* are summarised in Table 1.

## Discussion

In this study we investigated the possibility of using fluorescent molecular rotors for *in vivo* imaging of the microscopic viscosity, in a dynamic and quantitative manner. We demonstrated, for the first time, that microscopic viscosity can be measured in subcutaneous tumors *in vivo*, with subcellular resolution, using FLIM of viscosity sensitive water soluble molecular rotor BODIPY2.

It is known that abnormal levels of viscosity are associated with disease and malfunction. Viscosity changes in pathological cells or blood plasma have been shown, for example, for atherosclerosis^1^, hematologic disorders^4^, diabetes^3^, Alzheimer’s disease^7^, liver disfunction^3^, and cancer^40^,^41^,^42^.

Taking into account the worldwide expansion of oncological diseases, study of viscosity in neoplastic tissues deserves special attention. It should be noted that the published data on viscosity in cancer are quite contradictory, likely due to the fact that the measurements were performed “in bulk” and probably reflect an averaged viscosity over different parts of cells or tissues, as shown below. The first attempts to estimate the viscosity in cancer were made by Guyer *et al*. using the ultracentrifugation of cells suspensions. This study established that the relative viscosity of whole tumor cells is higher than that of normal cells and associated this phenomenon with accumulation of lactic acid in tumor^40^,^41^. Doblas *et al* used magnetic resonance elastography on cancer patients and demonstrated that the viscosity of malignant hepatic tumors was higher than that of benign lesions and significantly varied among the different tumor types^42^.

At the same time, the microscopic viscosity of individual domains of live cells *in vitro* was determined using fluorescence based methods, including molecular rotors^8^,^23^,^24^,^25^,^26^,^27^,^28^,^29^,^30^,^31^,^32^,^33^,^43^,^44^. Aqueous cytoplasm domain of cancer cells was reported to be less viscous compared with normal cells using radiofrequency electron paramagnetic resonance^45^. Rebelo *et al*. determined with atomic force microscopy that cancerous cells in culture are less viscous than non-tumorigenic cells^46^. In a model for progressive ovarian cancer using atomic force microscopy, Ketene *et al*. showed that mouse ovarian cells are more viscous when they are benign^47^.

The relationship between viscosity and chemoresistance of cancer cells was also investigated. It was revealed by Huang *et al*. with the use of fluorescence probe TMA-DPH *in vitro* that the plasma membrane microviscosity is higher in cells resistant to cisplatin^48^. Increased microviscosity was detected in plasma membranes isolated from cancer cells resistant to doxorubicine by staining with fluorescent probe pyrene^49^. It was also found by diffusion-time distribution analysis that microviscosity of plasma membrane of multidrug-resistant cancer cells is more heterogeneous in comparison with non-resistant ones^50^.

Moreover, the changes of cellular microviscosity during a light-induced cancer treatment termed Photodynamic Therapy (PDT) were reported. In PDT a drug termed photosensitiser is irradiated by visible light in the presence of oxygen causing death of malignant cells via the production of reactive oxygen species^51^. We have previously demonstrated using two independent molecular rotors that PDT of cells causes a large viscosity increase *in vitro*^30^,^31^. Furthermore, we have investigated the mechanism of this process using a third independent molecular rotor, BODIPY1, incorporated into model lipid bilayers^34^. In all cases we found that photooxidation of lipids or cellular components caused by PDT results in a large increase in viscosity.

Thus the literature data indicate that viscosity has the potential to be a biomarker for human malignancy and serve for prediction of tumor death due to PDT (and possibly other treatment modalities that induce cell death such as chemotherapy). However, until now the viscosity monitoring *in vivo* was not practically possible.

Here we report a protocol enabling such viscosity measurements in tumor bearing mice, over many hours following an injection of the fluorescent molecular rotor based on the BODIPY structure. BODIPY-based rotors were widely used previously for mapping viscosity on a single cell level *in vitro*^25^,^29^,^32^,^33^,^43^,^44^. Our present work makes it possible to use the same attractive approach *in vivo*, allowing quantitative viscosity determination, dynamic imaging in real time and good spatial resolution to be obtained. We resolve microscopic viscosity in individual tumor cells *in vivo* and confirm that the observed values are close to those obtained *in vitro* for the same tumor cell line.

Additionally, we measured the viscosity of collagen in CT26 tumor in mice using BODIPY1 dissolved in polymeric brushes. Synthetic organic nanoparticles are a promising alternative to liposomes and polymersomes, which allow to achieve high reproducibility of a formulation properties via controlled synthesis and excellent encapsulation of hydrophobic dyes^52^. This encapsulation avoids excessive binding to albumins in the blood (as observed in the case of liposomes, due to a high affinity of our probes to albumin^33^) and ensures the delivery of nanocarriers containing the rotor to tumours. Amphiphilic polymeric brushes used here are regular graft-copolymers of hydrophobic polyimide (PI) with hydrophilic polymethacrylic acid (PMAA) side chains forming particles that were about 100–150 nm in size^52^. The binding of BODIPY1 to polymeric brushes is due to hydrophobic interactions of the dye with PI and hydrogen bonding of the heterocyclic core with PMAA carboxyl groups. The size of the carriers is ideal for the EPR effect to the tumor vasculature. The pH sensitivity of PMAA^53^ can aid the cargo release in the acidic environment of the tumour.

Collagen, a major component of an extracellular matrix and the most important architectural element of a tumor tissue, plays a critical role in cancer progression. It is known that collagen is constantly degrading, redepositing, cross-linking and stiffening and as such is actively involved in regulation of tumor invasion, immune infiltration, metastasis, and angiogenesis^54^. However, little attention has been paid to biomechanics of this protein in the context of carcinogenesis. To the best of our knowledge, viscosity of tumor connective tissue has never been quantified before.

Our present report, therefore, provides a new methodology for *in vivo* monitoring of viscosity of tumors, which is likely to be useful for diagnostics and monitoring of the treatment progress and other therapeutic manipulation. The work to validate this method for PDT dosimetry and early evaluation of chemotherapy efficacy is currently underway in our laboratories.

## Materials and Methods

### General

BODIPY1^32^, BODIPY2^29^ and polymeric brushes^36^ were synthesised as reported previously. All solvents used for spectroscopic characterisation of rotors were spectroscopic grade. Quartz cuvettes with a 10 mm path length were used in all spectroscopic measurements. The concentration of BODIPY dyes was adjusted to the maximum absorbance of below 0.1 for all spectroscopic measurements to avoid reabsorption artefacts. Absorption spectra were measured using an Agilent 8453 UV-Vis spectrophotometer. Fluorescence spectra were recorded using a Fluoromax-4 spectrofluorometer (Jobin-Yvon; Horiba). The fluorescence decay traces in bulk samples were collected using a DeltaFlex Time-Correlated Single Photon Counting system (Horiba). The samples were excited at 467 nm using a pulsed NanoLED excitation source (IRF ca. 300 ps). Fluorescence was collected at 513 ± 10 nm. Decays were recorded until peak counts reached 10000 at a controlled temperature using a thermostatic circulating chiller (RE104, Lauda Technology Ltd.). Data were fitted to the appropriate exponential model after deconvolution of the instrument response function by an iterative deconvolution technique, using the IBH DAS6 fluorescence decay analysis software, where reduced (χ^2^) and weighted residuals serve as parameters for goodness of the fit.

### Cell culture

CT26 (murine colon carcinoma) cell line was used in the study. The cells were cultured in DMEM containing 100 μg/ml penicillin, 100 μg/ml streptomycin sulfate and 10% fetal bovine serum (FBS) at 37 °C in a humidified atmosphere with 5% CO_2_.

For microscopic imaging the cells were seeded on glass-bottom FluoroDishes in complete DMEM media without phenol red (Life Technologies). Before imaging, the culture media was replaced with ice-cold Hank’s solution without Ca^2+^/Mg^2+^, and cells were incubated at + 4 °C for 7 min. Afterwards, Hank’s solution was replaced with ice-cold BODIPY solution (4.5 μM, 0.1% DMSO).

The cells treated in a similar manner but without BODIPY, served as control.

We performed MTT assays of all probes at the working conditions used in this manuscript and we find no evidence of toxicity, Figure S7. The viability of cultured cells exposed to polymeric brushes was previously evaluated in^52^ and high cell viability was confirmed.

### Collagen gel preparation

In order to prepare three-dimensional collagen gel phantoms, 133 μL of 0.34 M sterile solution of sodium hydroxide was mixed with 200 μL concentrated (x10) culture medium 199, 8 μL glutamine, 70 μL 7.5% sodium bicarbonate and 40 μL HEPES. A cooled solution of type I collagen was added to this medium, and the mixture was placed on ice to prevent rapid gelation. At this stage a cell suspension of human skin fibroblasts was introduced into the mixture (20 000 cells per 1 mL). The resulting mixture was placed in glass-bottom FluoroDishes, and DMEM medium containing 100 μg/ml penicillin, 100 μg/ml streptomycin sulfate and 10% fetal bovine serum (FBS) was added and kept at 37 °C in a humidified atmosphere with 5% CO_2_. FLIM imaging of the cellularized collagen gel was performed after 3 days.

To measure viscosity of collagen, BODIPY solutions were added at a concentration of 45 μM, containing 0.5% DMSO. For better penetration of the rotors into the gel matrix, glass dishes were placed in a shaker for 3 hours at 22 °C before a microscopic investigation. To confirm the presence of collagen fibers, the second harmonic generation (SHG) signal was registered.

### Mice

Female BALB/c mice 10 weeks old, weighing 20–25 g were used. To generate tumors, the animals were challenged subcutaneously with 5 × 10^5^ CT26 (mouse colon carcinoma) cells in 100 μL PBS in the right flank. The experiments started 10–12 days after the cell injection, when the tumors had reached 7–8 mm in diameter. All experimental procedures conducted on animals were approved by the Ethical Committee of the Nizhny Novgorod State Medical Academy (Russia). All methods were carried out in accordance with relevant guidelines and regulations.

BODIPY1 and BODIPY2 were injected intravenously into the tail vain in doses 2–10 mg/kg. Tumor-bearing mice that did not receive BODIPY were used as controls. For imaging, animals were anesthetized with intramuscular injection of a mixture of Zoletil 100 (50 μL per animal, 40 mg/kg, Virbac SA, Carros, France) and 2% Rometar (10 μL per animal, 10 mg/kg, Spofa, Czech Republic).

### Fluorescence whole-body imaging

A molecular imaging system IVIS-Spectrum (Caliper Life Sciences, USA) was used for fluorescence whole-body imaging. Fluorescence of rotors was excited at a wavelength of 500/30 nm and detected at 540/20 nm. The images were acquired *in vivo* before the injection of rotor, after 15 min, 1, 2, 5 or 6 h, and 24 h. For imaging procedure, animals were anesthetized with 2% Isoflurane. The average fluorescence intensity (FI), (p/s/cm^2^/sr)/(μW/cm^2^), of each tumor was calculated at different time-points in Living Image 2.5 software, and corresponding value measured before the injection was subtracted.

### Biodistribution study

To analyze distribution of rotor in the animal body, fluorescence imaging *ex vivo* was performed. 24 hours after injection mice were sacrificed by cervical dislocation, tumor nodules and organs were immediately excised, washed with PBS and the fluorescence images were acquired on the IVIS-Spectrum system, as described above, and on the multiphoton tomograph MPTflex (JenLab, Germany). The average fluorescence intensity of tumor nodules and organs *ex vivo* was quantified from macroscopic images and normalized to corresponding values of the ones from CT26 bearing mice without any treatment.

### Plasma concentration analysis

To measure plasma drug level, BODIPY1 and BODIPY2 were injected intravenously to mice with CT26 tumor at the dose of 5 mg/kg and 10 mg/kg, respectively. Blood (20 μl) was collected from the retro-orbital sinus with a heparinized capillary tube after 5 min, l h, 2 h or 3 h, 4 h or 5 h, 6 h, 24 h and 48 h and centrifuged at 2500 rpm for 15 min to prepare plasma. Then 10 μL of the plasma was sampled, and dissolved in 2 mL of sterile saline. BODIPY fluorescence was analyzed by spectrofluorometry (Shimadzu RF-5301PC) (excitation at 475 nm, emission was scanned from 550–680 nm). The quantity of the rotors was determined by comparison of the relative fluorescence intensities at the wavelength of 510 nm with the calibration curves. To construct calibration curves, a known amount of the BODIPY was added in sterile saline.

### Multiphoton fluorescence microscopy and FLIM

Multiphoton tomograph MPTflex (JenLab, Germany) equipped with a tuneable 80 MHz, 200 fs Ti:Sapphire laser (MaiTai) and a TCSPC-based FLIM module (Becker&Hickl Inc., Germany) was used for multiphoton fluorescence microscopy and FLIM. The images were acquired through a 40x, 1.3 NA oil immersion objective.

BODIPY fluorescence was excited at the wavelength of 800 nm and detected in the range 409–680 nm. Autofluorescence in cells was excited at the wavelength of 750 nm and detected in the range 409–680 nm. SHG in collagen was exited at 750 nm and detected from 373 to 387 nm. The average power applied to the sample was ~ 12 mW. Image size was 512 × 512 pixels, and the acquisition time for one optical section was typically 7 seconds.

Two-photon excited (TPE) fluorescence and FLIM images of cultured CT26 cells were acquired within 5–10 min after adding BODIPY dyes.

For imaging, a skin flap over the tumor was surgically opened and the objective was placed directly on the tumor surface. TPE fluorescence, SHG and FLIM images were acquired every 20 min for 1.5 hours after an injection of BODIPYs and then again in 24 hours. Immediately after the imaging procedure, the skin flap was closed with 2–0 surgical suture.

### Fluorescence lifetime analysis

Fluorescence lifetime analysis was performed in the SPCImage software (Becker&Hickl Inc., Germany). Autofluorescence was shown to contribute insignificantly to the signal at 800 nm excitation. Time resolved fluorescence decays at each pixel of the whole image was fitted using a monoexponential model, and the fluorophore lifetime τ was calculated. The fluorescence lifetime distribution and the goodness of fit (χ^2^) histograms were analysed for each FLIM image. The χ^2^ ≤ 1.20 value for *in vitro* images and χ^2^ ≤ 1.40 for *in vivo* images indicated that the model used provided a reasonable fit.

The viscosity was correlated to the decay traces using the modified form of the Förster–Hoffmann equation in the logarithmic form: log τf = α log η + const. Using previously measured calibration plots for BODIPY1 and BODIPY2^17^ experimentally measured lifetimes (in ns) were converted to viscosity values (in cP).

### Statistical analysis

The mean values (M) and standard deviations (SD) were calculated for the long and short components of fluorescence lifetimes of BODIPY. The number of cells for mean value calculations was 20–30 in 7–10 fields of view.

## Additional Information

**How to cite this article**: Shimolina, L. E. *et al*. Imaging tumor microscopic viscosity in vivo using molecular rotors. *Sci. Rep.* **7**, 41097; doi: 10.1038/srep41097 (2017).

**Publisher's note:** Springer Nature remains neutral with regard to jurisdictional claims in published maps and institutional affiliations.

## Supplementary Material

Supplementary Information

## Figures and Tables

**Table 1:** Viscosity measurements *in vitro* and *in vivo* using BODIPY molecular rotors.

	BODIPY 1 (DMSO)	BODIPY 1 (i)	BODIPY 1 (ii)	BODIPY 2 (iii)
***IN VITRO***				
cancer cells	1.6 ns/160 ± 20 cP^32^,^33^	biexponential decay#	1.90 ± 0.05 ns/184 ± 11 cP	2.64 ± 0.09 ns/377 ± 27 cP
collagen	2.22 ± 0.06 ns/260 ± 15 cP	not detected	2.79 ± 0.15 ns/429 ± 51 cP	biexponential decay#
***IN VIVO***				
cancer cells	not detected	biexponential decay#	biexponential decay#	2.67 ± 0.06 ns/386 ± 19 cP
collagen	not detected	biexponential decay#	2.24 ± 0.06 ns/265 ± 16 cP	biexponential decay#

^#^Indicative of the dye aggregation, unsuitable for viscosity measurements.
