# Supplementary material for: Imaging tumor microscopic viscosity in vivo using molecular rotors
Source: Sci Rep. 2017 Jan 30;7:41097. doi: 10.1038/srep41097 (PMC5278387; doi:10.1038/srep41097)
Supplement: Supplementary Information [file srep41097-s1.doc]

Supplementary Information

**Imaging tumor microscopic viscosity in vivo using molecular rotors**

Lyubov’ E. Shimolina^1,2^, M. Angeles Izquierdo^3^, Ismael López-Duarte^3^, James A. Bull^3^, Marina V. Shirmanova^1^, Larisa G. Klapshina^4^, Elena V. Zagaynova^1^, Marina K. Kuimova^3^

1 – Institute of Biomedical Technologies, Nizhny Novgorod State Medical
Academy, Minin and Pozharsky Square, 10/1, Nizhny Novgorod, Russia 603005

2 - Institute of Biology and Biomedicine, Nizhny Novgorod State University,
Gagarin Avenue, 23, Nizhny Novgorod, Russia 603950

3- Department of Chemistry, Imperial College London, South Kensington, London SW7 2AZ, UK

4 - Razuvaev Institute of Organometallic Chemistry RAS, Tropinina Street, 49,
Nizhny Novgorod, Russia 603950

**Table of contents**

The accumulation and clearance of molecular rotors in vivo 3

Spectroscopic characterization of molecular rotors 4

Viscosity imaging in CT26 subcutaneous tumors in vivo 6

The accumulation of molecular rotors ex vivo 8

**The accumulation and clearance of molecular rotors *in vivo***

As control, we administrated BODIPY1 without polymeric brushes to mice bearing CT26 tumor in a dose of 8 mg / kg (5% DMSO). As shown in Figure S3, after an intravenous injection of the rotor fluorescence was not observed in the animal body.


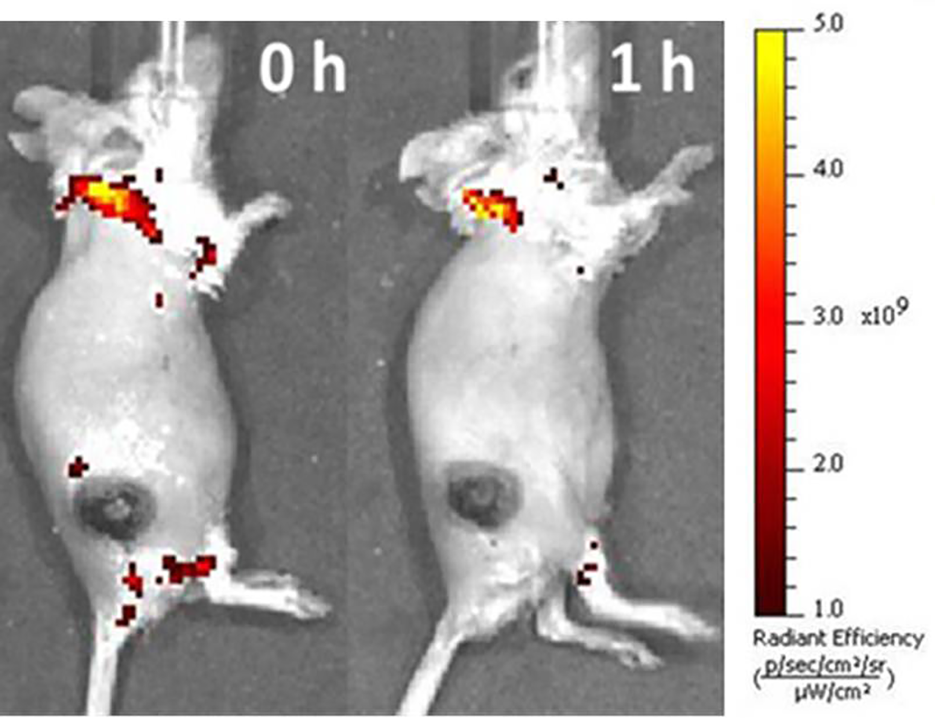


**Figure S1.** Fluorescence images of mice with CT26 tumor before and 1 hour after an i.v. injection of BODIPY1 without polymeric brushes (8 mg/kg; 5 % DMSO).

**Spectroscopic characterization of molecular rotors**

**
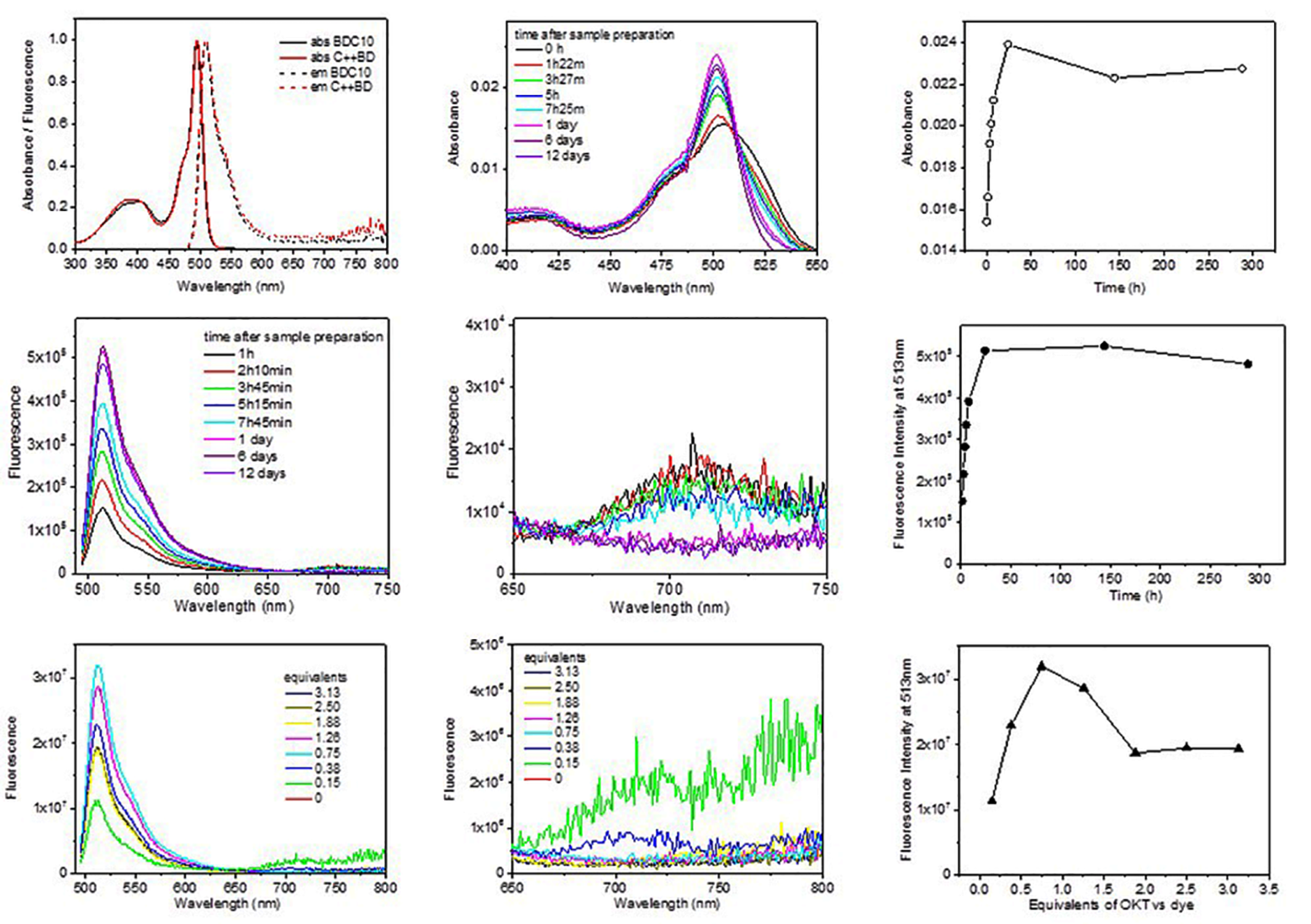
**

**Figure S2.** The photophysical characterisation of BODIPY1 and BODIPY2 with polymeric brushes in bulk solutions. (A) Normalized absorption (solid) and emission spectra (dash) of BODIPY1 (black) and BODIPY2 (red) in methanol. (B-F) Monitoring the timecourse of interactions of BODIPY1 with polymer brushes (1:1 molar concentration) using absorption (B, C) and fluorescence (D-I) spectroscopies; λ_exc_ = 467 nm. The narrowing of the main absorption band and an increase in the emission intensity is clearly observed as a function of incubation time in these solutions. (D-F) Monitoring the effect of the incubation time on the emission spectra, in the green spectral band (D, F) and in the red band corresponding to aggregated BODIPY species (E); (G-I) Monitoring the effect of the [Dye]:[brush] ratio on the emission spectra after 24h incubation, in the green spectral band (G, I) and in the red band corresponding to aggregated BODIPY (H). From these data we conclude that the optimal conditions that allow full disaggregation, high emission (D, G) and the absence of aggregates (E, H) of BODIPY1 are: 24h incubation (C, F) and 1:1 [BODIPY1]:[brush] (I).

**
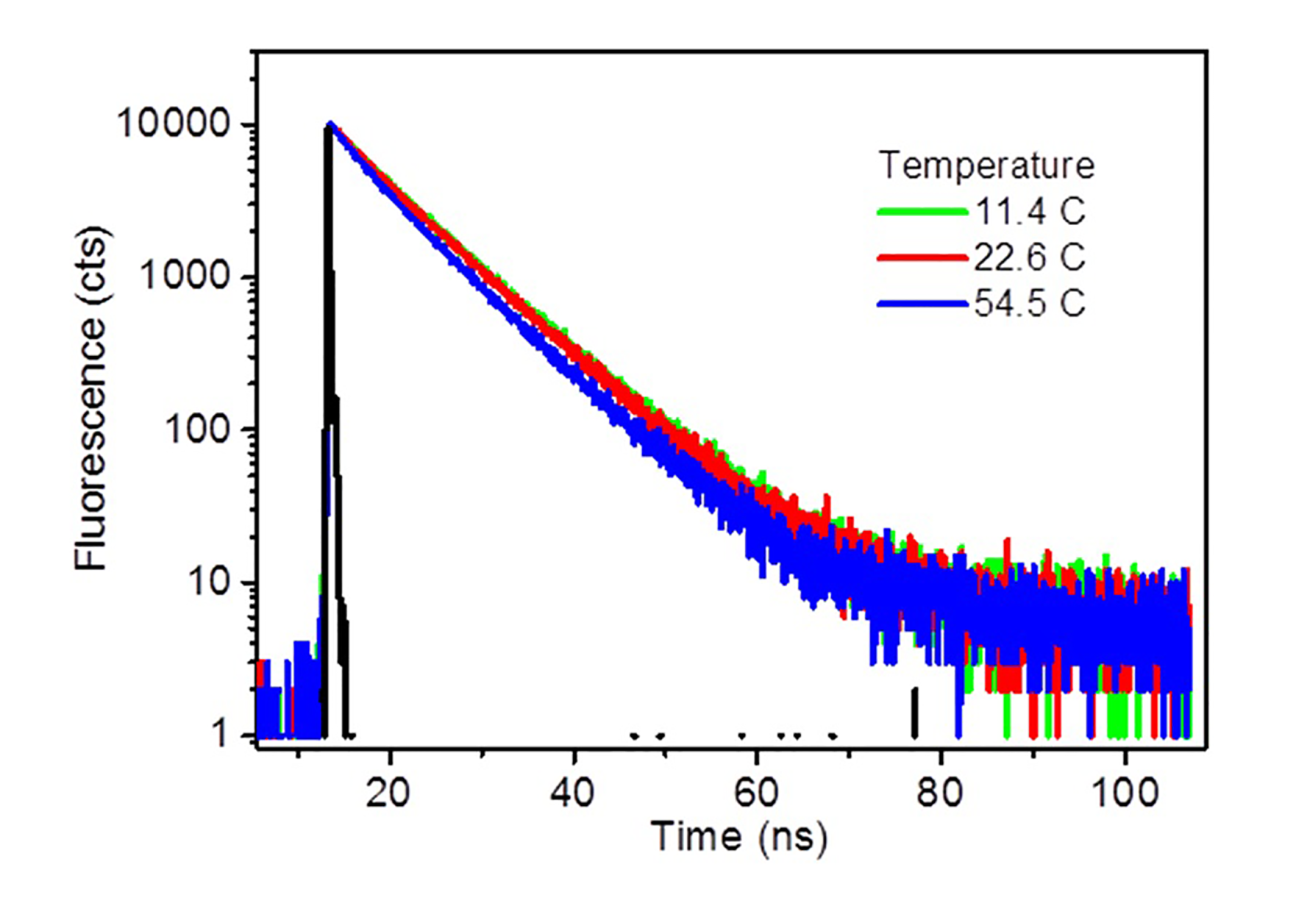
**

**Figure S3.** Time-resolved fluorescence decay traces recorded for BODIPY1 in the presence of 1 equivalent of a polymeric brush at different temperatures; λ_exc_ = 467 nm, λ_em_ = 513±10 nm. The decay at 22^◦^C can be fitted with the following parameters
τ_1_ = 6.55 ns (65%), τ_2_ = 10.01 ns (35%), χ^2^ = 1.42.

**Viscosity imaging in CT26 subcutaneous tumors *in vivo***


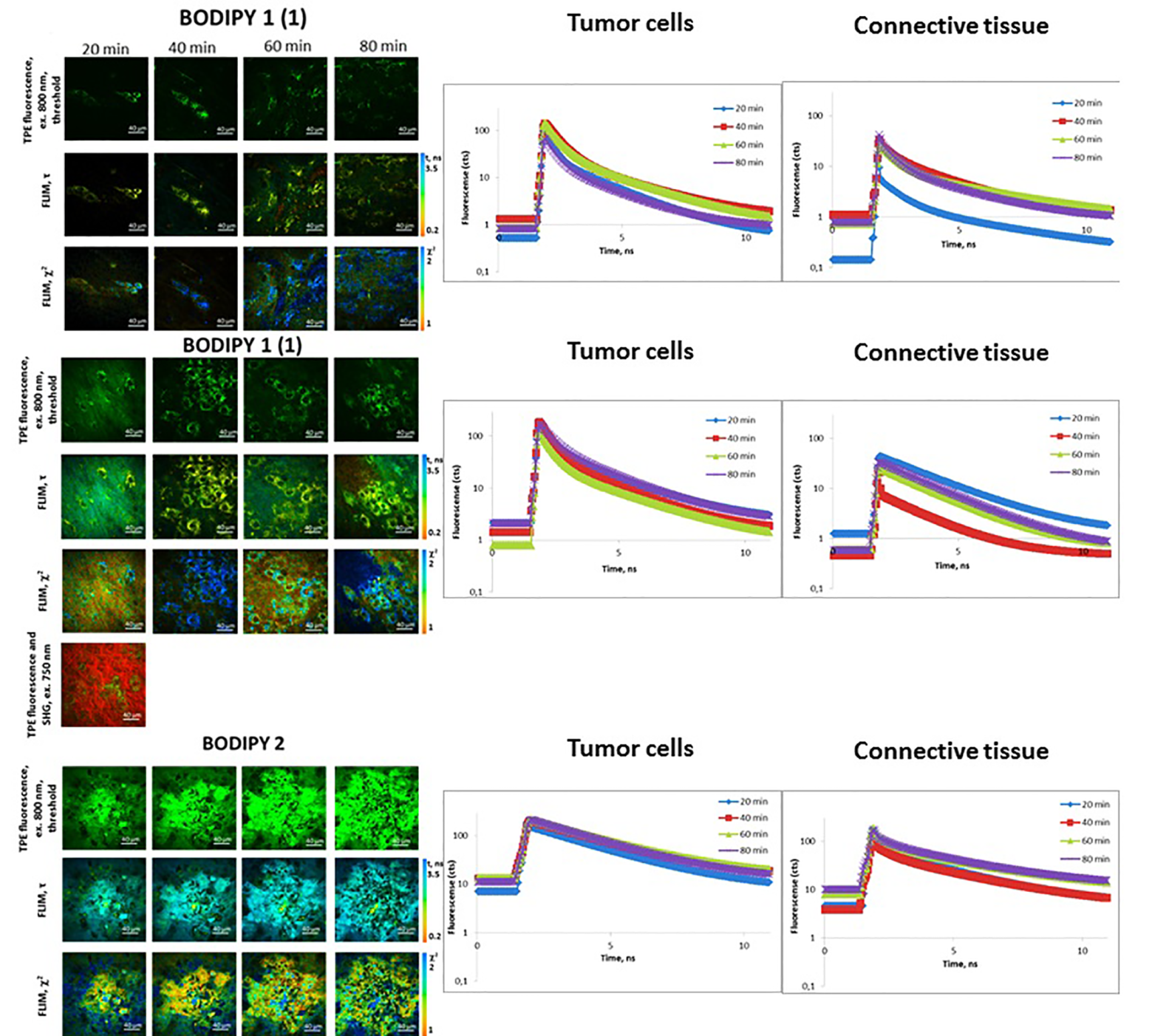


**Figure S4.** The dynamics of the distribution of the rotors over time in tumor tissue after an i.v. injection of BODIPY1 in a dose of 3 mg/kg dissolved in polymeric brushes (8 and 14.8 mg/kg), corresponding to labels (1) and (2), respectively); and after an i.v. injection of BODIPY2 in a dose of 3 mg/kg. Two-photon excited fluorescence, FLIM lifetime maps, typical time resolved decay traces and FLIM χ^2^ maps are shown, excitation was 800 nm, detection 409-680 nm. In order to obtain SGH images 750 nm excitation was used.

The FLIM χ^2^ maps confirm good monoexponential fitting in the areas where χ^2^≤1.5 (indicated by the orange to green colour).

More specifically, BODIPY1 (1) shows biexponential decays both in cells and in connective tissues. For tumor cells the averaged decay from the field of view containing many cells can be fitted with the following parameters t1=0.48 ns (80%), t2 = 2.18 ns (20%), χ^2^=1.13;
for connective tissues - t1=0.73 ns (70%), t2 = 2.92 ns (30%), χ^2^= 0.90,

BODIPY1 (2) shows biexponential decays in tumor cells; the averaged decay from the field of view containing many cells can be fitted with the following parameters: t1=0.67 ns (75%), t2 = 2.60 ns (25%), χ^2^=1.05.

However, monoexponential decays were observed in connecting tissues (t=2.24 ns (100%), χ^2^=1.32).

BODIPY2 shows biexponential decays in connecting tissues, the averaged decay from the field of view containing many cells can be fitted with the following parameters: t1=0.45 ns (45%), t2 = 2.94 ns (55%), χ^2^=1.10 and monoexponential decays in tumor cells (t = 2.67 ns (100%), χ^2^=1.30).

**The accumulation of molecular rotors *ex vivo***

The study of the BODIPY1 distribution *ex vivo* showed that 24 hours after intravenous injection, the rotor accumulated to a large degree in the liver, colon and skin.


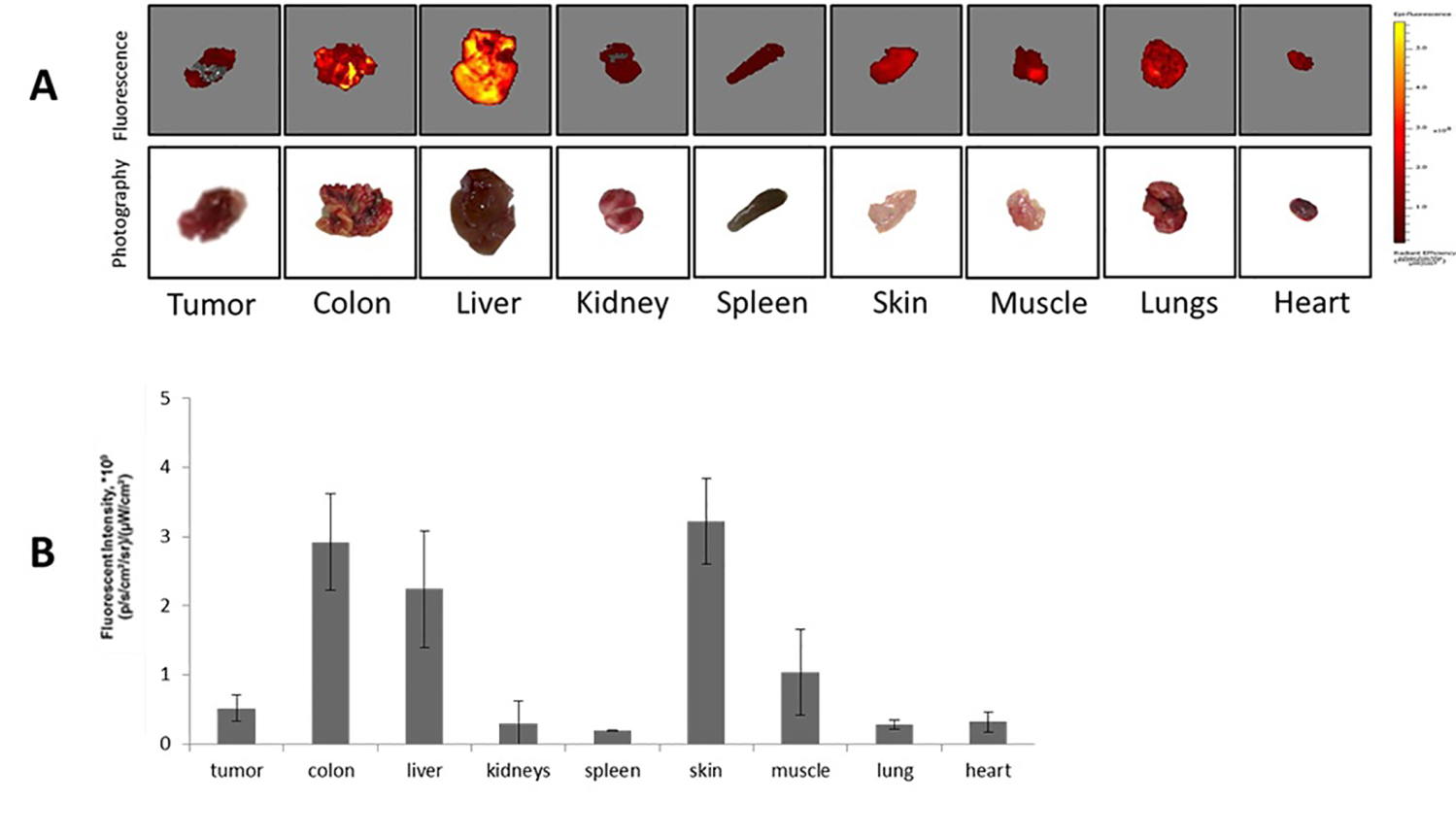


**Figure S5.** Representative ex vivo fluorescence images of the tumor and organs at 24 hours (A) and quantification of the fluorescence signals (B) after an i.v. injection of BODIPY1 (1) at a dose of 5 mg/kg dissolved in polymeric brushes (at 12 mg/kg) into CT26 bearing Balb/c mice. Mean ± SD (n=3).

While BODIPY2 24 hours after intravenous administration was detected to a large degree in the liver, colon, kidney, and skin.

**
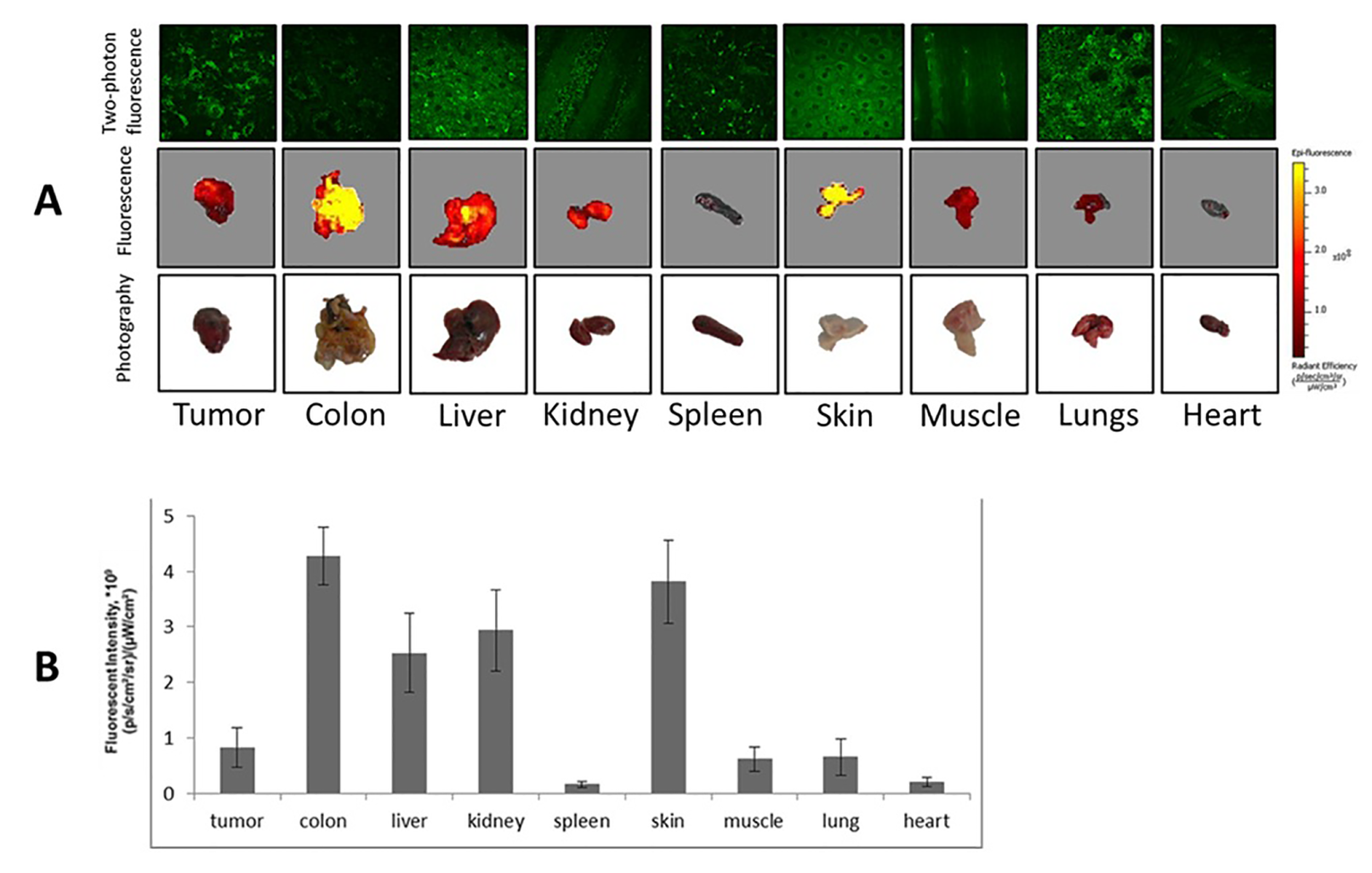
**

**Figure S6.** Representative ex vivo fluorescence images of the tumor and organs at 24 hours (A) and quantification of the fluorescence signals (B) after an i.v. injection of BODIPY2 at a dose of 10 mg/kg into CT26 bearing Balb/c mice. Mean ± SD (n=3).

We performed MTT assays of BODIPY 1 (DMSO) and BODIPY2 (iii) at the working conditions used in this manuscript and we found no evidence of toxicity


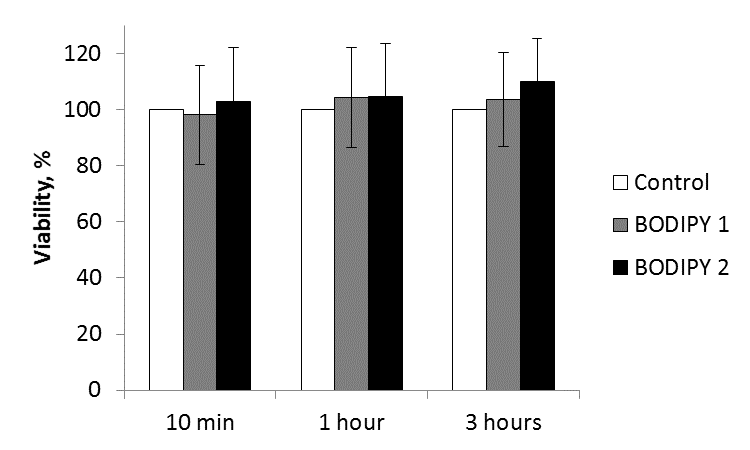


**Figure S7.** Cell viability of CT26 cells treated with BODIPY 1 (DMSO) (A) and BODIPY2 (iii) (B) in the dark. CT26 cells were incubated with 4.5 µM solutions of BODIPY1 and BODIPY2 and MTT assay was performed at 10 min, 1 hour and 3 hours of incubation. Error bars correspond to standard deviations from 15 separate measurements.
